# Twinning during the pandemic: Evidence of selection *in utero*

**DOI:** 10.1093/emph/eoab033

**Published:** 2021-10-20

**Authors:** Ralph Catalano, Tim Bruckner, Joan A Casey, Alison Gemmill, Claire Margerison, Terry Hartig

**Affiliations:** 1 School of Public Health, University of California, Berkeley, Berkeley, CA, USA; 2 Program in Public Health and Center for Population, Inequality and Policy, University of California, Irvine, Irvine, CA, USA; 3 Environmental Health Sciences, Columbia University, New York, NY, USA; 4 Johns Hopkins Bloomberg School of Public Health, Baltimore, MD, USA; 5 Department of Epidemiology & Biostatistics, Michigan State University, East Lansing, MI, USA; 6 Institute for Housing and Urban Research, Uppsala University, Uppsala, Sweden

**Keywords:** COVID-19, twins, selection *in utero*

## Abstract

**Background and objectives:**

The suspicion that a population stressor as profound as the COVID-19 pandemic would increase preterm birth among cohorts in gestation at its outset has not been supported by data collected in 2020. An evolutionary perspective on this circumstance suggests that natural selection *in utero*, induced by the onset of the pandemic, caused pregnancies that would otherwise have produced a preterm birth to end early in gestation as spontaneous abortions. We test this possibility using the odds of a live-born twin among male births in Norway as an indicator of the depth of selection in birth cohorts.

**Methodology:**

We apply Box–Jenkins methods to 50 pre-pandemic months to estimate counterfactuals for the nine birth cohorts in gestation in March 2020 when the first deaths attributable to SARS-CoV-2 infection occurred in Norway. We use Alwan and Roberts outlier detection methods to discover any sequence of outlying values in the odds of a live-born twin among male births in exposed birth cohorts.

**Results:**

We find a downward level shift of 27% in the monthly odds of a twin among male births beginning in May and persisting through the remainder of 2020.

**Conclusions and implications:**

Consistent with evolutionary theory and selection *in utero*, birth cohorts exposed *in utero* to the onset of the COVID-19 pandemic yielded fewer male twins than expected.

**Lay Summary:**

Our finding of fewer than expected male twin births during the onset of the COVID-19 pandemic provides more evidence that evolution continues to affect the characteristics and health of contemporary populations.

## INTRODUCTION

The large literature reporting that pregnant women in stressful environments contribute more than their proportional share of preterm births leads to the suspicion that the incidence of such births increased among infants born in 2020 when many human populations experienced the somatic and psychosomatic stressors of the COVID-19 pandemic [1]. The data currently available from 2020 do not, however, confirm that suspicion. No clear association appears between SARS-CoV-2 infection and preterm birth at either the individual or population level [2]. Explanations of these counterintuitive findings include the argument that many of the pregnancies which would have produced, absent the pandemic, a preterm birth in 2020 ended early in gestation as spontaneous abortions given the unusually high dose of stressors visited upon human populations [3]. This argument has roots in the behavioral ecology literature that invokes reproductive suppression [4], brood reduction [5, 6] and offspring sex selection [7] to refer to conserved mechanisms that avert parental investment in offspring with poor fitness prospects. Among humans these mechanisms presumably include selection *in utero* [8].

Selection *in utero* assumes that natural selection conserved mutations that protect maternal reproductive fitness by detecting and aborting fetuses least likely to produce grandchildren [8]. Such mechanisms would abort fetuses signaling, e.g. chromosomal or genetic abnormalities. The literature invokes selection *in utero* to explain the changing ratio of male to female fetal loss over the course of gestation as well as differences in the ratio among birth cohorts observed over time and across space [9, 10].

Female fetuses in the first 2 months of gestation exhibit ‘oogonia’ that, via meiosis, develop into the oocytes of all potential offspring [11]. Meiosis, however, can produce chromosomal errors causing a female fetus to signal not only its own genetic and chromosomal defects (i.e. those of the pregnant woman’s prospective daughter) but also those of its children (i.e., the grandchildren of the pregnant woman) [12–14]. A female fetus provides, in effect, information describing reproductive fitness of two generations of the pregnant woman’s descendants, whereas a male conveys information for only one [13, 15]. Consistent with the assumption that this information emerges with the development of oogonia, selection early in gestation appears greater against female than male fetuses [16].

Although male, like female, fetuses that signal chromosomal and genetic defects suffer spontaneous abortion early in gestation, small but otherwise ‘normal’ males spontaneously abort later in gestation more frequently than similarly small but normal females [17]. As a result, the ratio of male to female spontaneous abortions exceeds one in clinically recognized (i.e., after about 8 weeks of gestation) pregnancies [18]. The theory of selection *in utero* attributes this high ratio to the fact that males small at birth produce fewer offspring than do other males [19]. This relatively low reproductive fitness presumably arises, at least in part, from the relative frailty of small male infants. For every society and year for which we have dependable life table data, male infants more likely die than any other age-by-sex grouping from birth through the end of reproductive life [20]. Epidemiology shows, moreover, that small size predicts death more strongly among male than female infants [21, 22]. Research further reports that size needed to avoid spontaneous abortion varies not only among pregnant women but also with the dose of stressors imposed on the population [8, 21].

The above literature suggests that natural selection has conserved mutations that spontaneously aborted small male fetuses when the environment turns threatening to either maternal resources or infant survival [22]. The theory of selection *in utero* assumes that this set of conserved mechanisms continues to operate in contemporary conception cohorts [9].

The few peer-reviewed tests of the association between the sex ratio of spontaneous abortions and stressful environments have supported selection *in utero* [23, 24]. The reliability of these tests remains, however, difficult to assess. Differences over time and space in requirements for, and the effort invested in, registration of spontaneous abortions induce artifactual variation in the data as do temporal and spatial differences in the efficacy of clinical interventions intended to ‘rescue’ fetuses at risk of such abortions [25].

As an alternative to registered spontaneous abortions, researchers have used the sex ratio of live births (i.e. the secondary sex ratio) to gauge the depth of selection *in utero* in birth cohorts. Empirical tests of the association between population stressors and the secondary sex ratio frequently report that the ratio decreases during stressful times [8]. An issue with this approach arises, however, from the fact that a ratio can vary over time as much from changes in the denominator as in the numerator. Because observational data suggest that females prevail among all spontaneous abortions [16], the question arises whether the fate of male or female fetuses accounts for temporal and spatial variation in the secondary sex ratios.

Other research has used the frequency of twins among male live births as an indicator of the depth of selection in birth cohorts because gestations yielding a male twin have historically produced the fewest grandchildren per pregnancy [26, 27]. This low reproductive fitness arises, in part, because male twins die more frequently in infancy than do other male, and all female, infants [28]. The low fitness of gestations that include a male twin may also reflect the fact that *females* in gestation with a male twin have fewer children than other females [29]. Whatever its cause, the low reproductive fitness of gestations including a male twin makes them a likely target of selection *in utero—*particularly during stressful times.

The epidemiologic and demographic literature includes reports consistent with the argument that selection *in utero* targets twins in general and male twins in particular [10]. Twins represent an estimated 12% of human conceptions but ∼2% of pregnancies that yield live births [30]. About 30% of clinically detected twin pregnancies convert, *via* the spontaneous abortion of a fetus, to singleton deliveries [30]. Males, moreover, appear to predominate among clinically detected spontaneously aborted twins [31, 32]. And singleton males who survive a gestation from which a male twin spontaneously aborted suffer growth retardation more frequently than other singleton males [31, 32]. As noted above, growth-retarded males have fewer children than males born larger [21].

More relevant to our test, studies of the frequency of twins among male births in Scandinavia report, consistent with selection *in utero*, that the odds of a male twin drop during stressful times [33–35]. This association, unlike that between stressful times and spontaneous abortion, does not arise from poor ascertainment because no controversy surrounds the registration of twin births. And, unlike the secondary sex ratio, no ambiguity arises over which sex accounts for temporal or spatial variation in the sex-specific odds of twins.

We exploit strong theory and data from Norway to test the hypothesis that the monthly odds of a twin among male births from March through November 2020 exhibited a sequence of detectably lower than expected values. We also test the hypothesis that any sequence of detectably lower than expected monthly odds of a twin among female births will include fewer cohorts than among male births. And last, because selection *in utero* late in gestation appears primarily against males, we also test the hypothesis that any detectable sequence of lower than expected odds of a twin among male births will appear in cohorts born earlier in time after the onset of the pandemic than any drop in the odds among female births.

We use Norwegian data for the following reasons:

1. The epidemiologic literature reports that Norwegians suffered significant increases in anxiety and stress during the early weeks of the pandemic. Blix *et al*. [36] found that, in May 2020, 25.7% of Norwegians reported symptoms of psychological distress above the level thought clinically significant. By comparison, only 14% reported symptoms above that level in 2019. Blix *et al*. further report that women appeared significantly more likely than men to report pandemic-related anxiety and depression. Similarly, Ebrahimi *et al*. [37] reported that, early in the pandemic, 27.57% (95% CI = [26.70%, 28.43%]) of Norwegians reported clinically significant anxiety—a level two to three times higher than estimates using the same instrument from similar samples in pre-pandemic periods. A larger proportion of women than of men met the criteria for clinically significant depression and anxiety. We have no reason to believe that pregnant women experienced less distress than did Norwegian women overall.
2. The research, alluded to above, reporting low reproductive fitness among gestations yielding male twins [26, 27] used Scandinavian data as did the study of low reproductive success among males small at birth [21]. Only Norway among Scandinavian countries had made 2020 twin births by month publicly available at the time of our data search (April 2021).
3. Earlier research in Norway has reported that the frequency of twins among male births dropped after an unusual population stressor (i.e. the murders of 77 Norwegians, including many teenagers, perpetrated in a single day by Anders Breivik in 2011) [35].
4. Norway’s response to the COVID-19 pandemic has been well-documented as swift and comprehensive. We, therefore, know when and how the everyday life of Norwegians changed by virtue of the pandemic. In the interests of brevity, and because Norway’s response has been described in detail elsewhere [38], we only summarize those changes here. On 12 March 2020, Norwegians began, by law, to distance themselves from each other by reducing all but necessary contact. Social gathering whether in homes or commercial establishments stopped. Schools closed. Businesses furloughed non-essential employees. Many workers who remained employed worked from home, often with children who needed additional help with school tasks. Use of non-essential health care stopped but prenatal care remained available for women advised, or who chose, to use it. In sum, although the onset of a virulent pandemic did not deny pregnant Norwegian women access to care, it started a cascade of changes in everyday life that for many people included additional stressors, reduced access to social coping resources, and constrained opportunities for psychological restoration.

## METHODS

### Data and variables

We test our three hypotheses using data from Norway before and during the COVID-19 pandemic. We acquired monthly sex-specific counts of singleton and twin births in Norway from the Medical Birth Registry of Norway. We calculated our dependent variable as the natural logarithm of the monthly odds of a twin among live male and, separately, female births in Norway for the 59 months starting January 2016 and ending November 2020. We transformed the sex-specific odds to their natural logarithms to allow us to express findings as percent of monthly expected odds represented by observed values.

We used 59 monthly cohorts because this provided 50 pre-pandemic cohorts with which to ‘train’ the model that predicted counterfactual values for the 9 intra-pandemic test monthly cohorts. Fifty monthly cohorts suffice to efficiently model autocorrelation, including seasonality, while avoiding the exploitation of higher counts to shrink detection intervals [39].

The denominator for our odds included singletons born in the same and subsequent month as twin births. We included singletons in the subsequent month because many twins are born before 36 complete weeks of gestation implying that the conception cohort at risk of producing a twin in month *t* includes infants born in months *t* and *t* + 1. This formulation of the population at risk precluded using births in December 2020 in our test because we do not have birth data from January 2021.

The relatively low levels of infection and hospitalization enjoyed by Norwegians allowed the easing of some restrictions as early as mid-April 2020. The greatest dose of stress on the population, therefore, likely occurred from mid-March through mid-April 2020. Although life did not return to ‘normal’ in mid-April, the nature of the threat and of the means to cope with it appeared better understood. For the purposes of our test, we therefore characterized the cohorts born from March through November 2020 as exposed to deeper than expected selection *in utero*. Live-born twins exposed earliest in gestation to pandemic-related restrictions would have been conceived in March 2020 and born in October and November. Those exposed latest in gestation would have been conceived in July and August 2019 and born in March 2020.

### Analyses

Measuring the pandemic’s association, if any, with the odds of a twin birth requires counterfactuals, or estimates of the odds had the pandemic not occurred. An intuitive approach to devising counterfactuals treats pre-pandemic birth cohorts as representative of cohorts born during the pandemic. The statistically expected value of the sex-specific odds of a twin in pre-pandemic cohorts therefore serves as the counterfactual for intra-pandemic cohorts.

The logic of the above approach seems unassailable, but investigators often assume that the mean of an outcome’s pre-pandemic values is its expected value and can, therefore, serve as an intra-pandemic counterfactual. This assumption does not, however, apply if the outcome in pre-pandemic cohorts exhibits patterns over time. Such patterns, or ‘autocorrelation’, imply that the expected, or counterfactual, values of a series of measurements are not their mean, but extrapolations or forecasts from their history.

We devised our counterfactuals with Box–Jenkins methods widely used in engineering and in the natural as well social sciences to systematically detect and mathematically model autocorrelation [40]. Patterns detected by these methods include trends induced by, e.g. increased use of *in vitro* fertilization [41]. Other detected patterns include cycles that in monthly data typically indicate strong seasonality. Most importantly, the methods also identify autocorrelation that becomes statistically detectable after a high or low value. These patterns include autoregression or ‘echoes’ in which high or low values are followed either immediately or later by similarly high or low, but geometrically declining, values. Other patterns appear as moving averages or ‘plateaus’ in which a high or low value persists for one or more cohorts and then drop or rise abruptly back to previous levels. Such ‘plateaus’ can be in the same or opposite direction as the original high or low value and may appear not only immediately but also later in the time series.

Our analyses proceeded through the following steps. First, we used Box–Jenkins methods to detect autocorrelation in the natural logarithms of the sex-specific odds of a twin birth in Norway for the 50 months starting January 2016 and ending February 2020. Second, we used the two models of autocorrelation detected in Step 1 to forecast values for 9 additional months of 2020 (i.e. the first 9 pandemic months). Third, we devised our counterfactual series for each variable by joining the fitted values from the models estimated in Step 1 with the 9 forecasts. Fourth, we estimated ‘other-than-expected’ values by subtracting the counterfactual series from the observed data. Fifth, we used the methods of Alwan and Roberts [42] to detect outlying sequences in the last 9 months (i.e. March through November) of the other-than-expected values. These methods detect high or low deviations from the expected odds of twinning among males. These deviations can appear as single ‘spikes’, spikes followed by geometrically declining values, or level shifts. We set the detection interval for outliers at 99.5% to reduce the likelihood of false rejection of the null.

Results would support our first hypothesis that the odds of a twin among male births fell in birth cohorts in gestation during March 2020, if we detected a negatively signed outlying sequence in the last 9 of the other-than-expected values. Results would support our second hypothesis if any outlying sequence in the last 9 other-than-expected values for females included fewer birth cohorts than any low sequence detected among males. And, last, results would support our third hypothesis if any outlying sequence detected for males appeared earlier among the last 9 other-than-expected values than any outlying sequence detected for females.

## RESULTS

Table 1 shows the means and range for sex-specific singleton and twin births in Norway for 59 test months from January 2016 through November 2020. Table 1 also shows the mean and range of our dependent variable—the natural logarithms of the monthly odds of a twin among Norwegian male and female live births.

**Table 1.** Means and range of sex-specific monthly singleton and twin births (and of the monthly sex-specific logged odds of a twin birth) in Norway for January 2016 through November 2020

	Singletons		Twins		Logged odds of a twins	
	Males	Females	Males	Females	Males	Females
Minimum value	1825	1754	39	35	−4.73	−4.80
Maximum value	2827	2569	98	95	−3.90	−3.87
Mean	2350	2217	70	68	−4.21	−4.19

**Figure 1.**
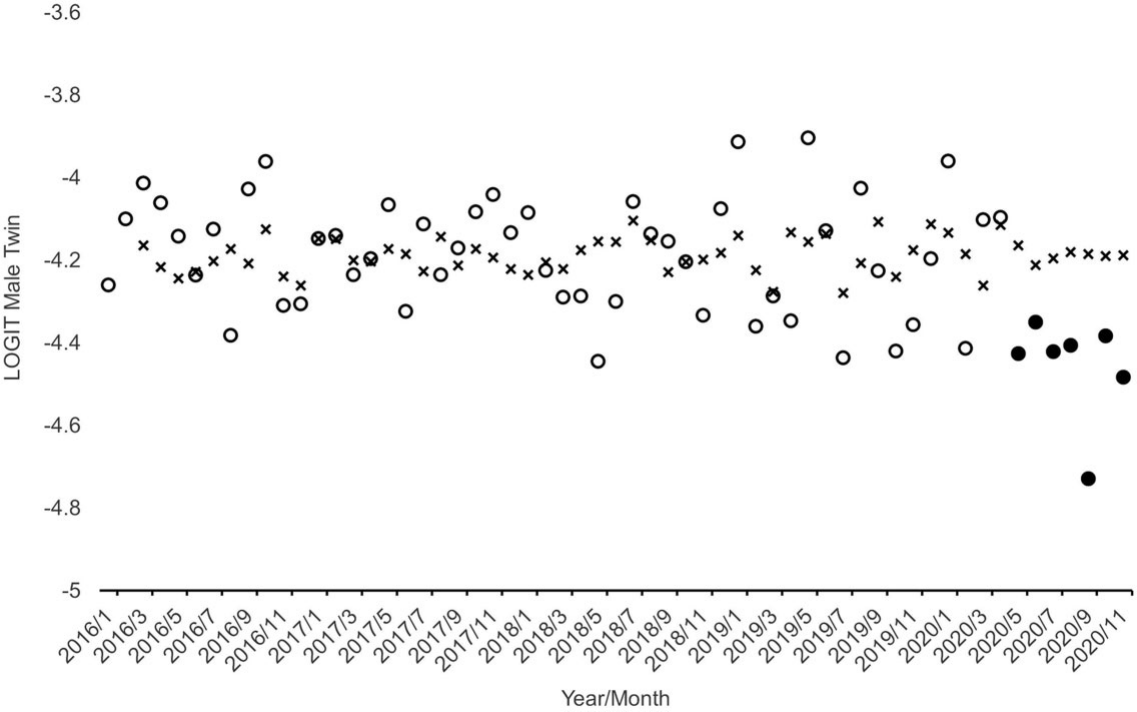
Observed (circles) and expected (x’s) monthly logged odds of a twin among male infants born in Norway from January 2016 through November 2020. Filled circles show outlying sequence

**Figure 2.**
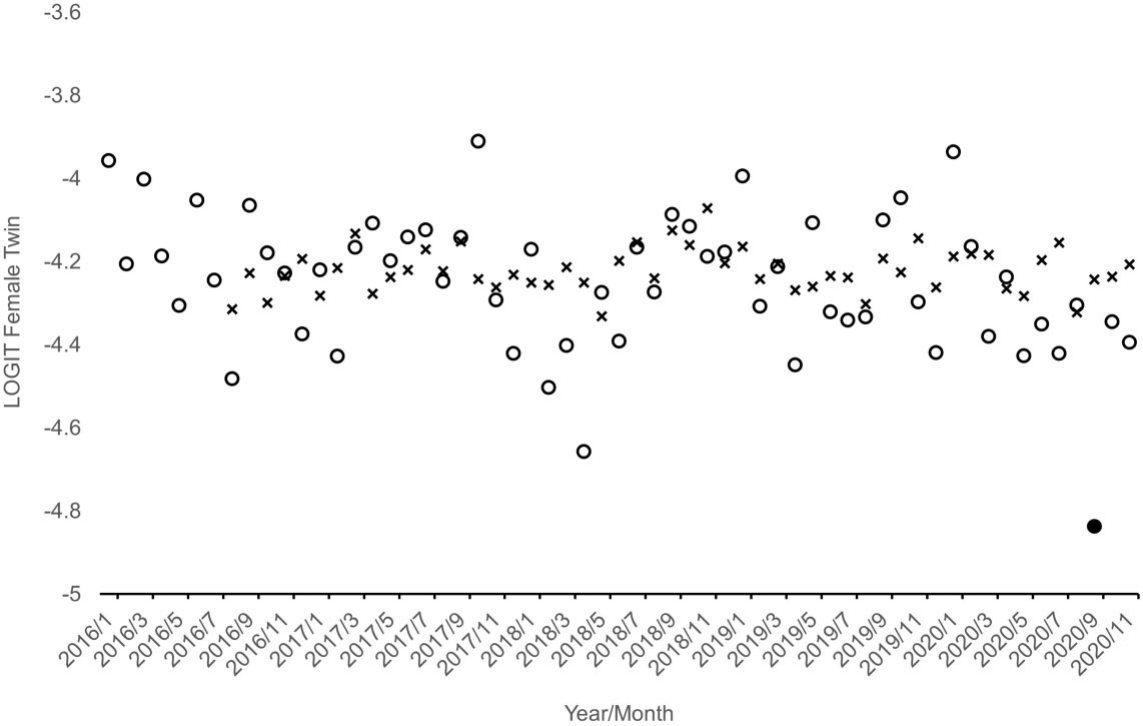
Observed (circles) and expected (x’s) monthly logged odds of a twin among female infants born in Norway from January 2016 through November 2020. Filled circle shows outlying value

Figures 1 and 2 show, as circles, the natural logarithms of the sex-specific odds plotted over the 59 test months.

Using Box–Jenkins methods to detect and model autocorrelation in the natural logarithms of the pre-pandemic odds shown in Figs 1 and 2 yielded the following models:

$$\text{Males}\;{\left[mt_{t}/ms_{t}\right]}^{e}=-4.187+1/\left(1-0.324B^{2}\right)a_{t}$$

$$\text{Females}\;{\left[ft_{t}/fs_{t}\right]}^{e}=-4.222+1/\left(1-0.{347B}^{7}\right)a_{t}.$$

In which [*mt_t_*/*ms_t_*]^*e*^ and [*ft_t_*/*fs_t_*]^*e*^ are the natural logarithms of the odds of a twin male or female birth in month *t*. −4.187 and −4.222 are constants. −0.324 and −0.347 are autoregressive parameters implying that a high or low value in the logged odds of a twin among male and female births exhibited detectable autocorrelation in the form of ‘echoes’ 2 and 7 months later, respectively. *a_t_* are the error terms at month *t*. Constants and autoregressive parameters all exceeded twice their standard errors.

As noted above, to satisfy the assumptions of statistical estimation of association, time-series modeling removes all forms of autocorrelation—whether or not hypothesized *a priori*—from the dependent variable before measuring its association with the ‘interruption’ (mandated social distancing in our case). Our test, therefore, ‘controls’ for autoregression at 2 and 7 months for males and females respectively even though we did not predict this autocorrelation *a priori*.

Figures 1 and 2 also show, as x’s, our counterfactual series formed in Steps 2 and 3 in which we joined the fitted values from the models shown above with nine forecasts from each model. The last nine x’s in each figure show the forecasted log odds for March through November 2020. 

Figures 1 and 2 show, as filled circles, the sequences detected by Alwan and Roberts methods, applied in Steps 4 and 5 in the analysis, as outside the 99.5% detection interval. As hypothesized, the odds of a twin among male births fell below the interval in May and remain an average of 27% below expected for the remainder of the series (i.e. for seven cohorts). Consistent with our second hypothesis, only one among the nine exposed cohorts exhibited odds of a female twin below the 99.5% detection interval. Consistent with our third hypothesis, the one detectably low cohort of female twins appeared in September, 4 months after the detectable decline began among male cohorts.

## DISCUSSION

We find evidence that, consistent with theory, the onset of the COVID-19 pandemic and the steps taken to avert its worst effects, may have deepened selection *in utero* in Norway. If so, the unexpectedly low rates of preterm birth observed in 2020 may reflect, at least in part, spontaneous abortion of fetuses that in more benign times would have survived to live birth later in pregnancy but before 36 complete weeks of gestation.

Norway experienced relatively low rates of SARS-CoV-2 infection and excess mortality during our test period, making it a good setting in which to estimate the association between social processes, as opposed to infection, and twin births. The pandemic could have engendered stress not only by threatening and causing infection, but also by imposing demands on individuals and households (e.g. caring for children out of school or ill relatives) while also disallowing access to social resources (e.g. supportive co-workers), and constraining access to settings (e.g. pubs, restaurants, entertainment events, gyms, athletic fields, second homes) that Norwegians would otherwise rely on for effective restoration. We, however, have no measurements of access to social and other coping resources in Norway during the pandemic. We cannot, therefore, estimate whether an increase in infection or a decrease in protective or restorative resources better ‘fits’ the drop in twin births that we found. We note, though, that previous studies with Norwegian and Swedish data have found indications of higher rates of spontaneous abortion under stressful circumstances that do not involve infection [35, 43–45]. Constrained opportunities to adequately restore psychological and other adaptive resources needed to meet the demands of everyday life appear, moreover, associated with indicators of selection *in utero* [46]. The pandemic management strategy of reducing social contact would seem to constrain opportunities for restoration. Working from home, e.g. could have reduced the restorative benefits of homelife that would otherwise have helped people maintain the psychological and relational resources they needed to meet the demands of life under pandemic circumstances [47].

We further note that pandemic management strategies may have reduced the dose of a least one ambient stressor, traffic-related air pollution (e.g. nitrogen dioxide) [48], thought to induce spontaneous abortion. In Norway, 2020 levels of NO_2_ dipped nearly 10 μg/m^3^ below pre-COVID-19 levels [49]. Our findings imply that the virulence of social and psychological stressors induced by the pandemic outweighed any benefit attributable to improved environmental conditions.

Limitations of our tests include that we cannot know the gestational age, or ages, at which twins *in utero* appeared most susceptible to the COVID-19 pandemic. Data on gestational age of live births and spontaneous losses would allow the creation of a time series arrayed by estimated month of conception (rather than by month of birth). Such series would allow identifying which conception cohorts contributed most to our birth cohort findings. That information would, in turn, suggest the gestational age at which stressors on the population trigger selection *in utero* against male twins.

Pandemics, by axiom, affect most human populations. That fact, and the lack of twin birth data from other societies, precludes our using a comparison population design to control for generally occurring confounders [50]. The pervasive and profound effect of the pandemic on everyday life; however, makes it unlikely that our findings arise from a powerful population stressor unrelated to, but entirely coincident with, the pandemic.

Interventions in Norway intended to increase social distancing early in the pandemic included suspending medically assisted reproduction (MAR) services [51]. Twins, therefore, could appear less frequently among cohorts conceived from mid-March through April 2020 because gestations begun in assisted reproduction clinics produce more than their proportional share of twins. Although the curtailment of MAR does not affect our test, which focused on cohorts already in gestation in March 2020, it would complicate extending the test to include gestations conceived early in the pandemic. Research into the implications of the pandemic for the conception of twins will have to sort out the contribution of MAR not only to the frequency of twin conceptions but also to the sex ratio of twins [52–54]. This sorting out would likely require knowing the fraction of twin births contributed by monozygotic gestations as well as the mix of male–male, female–female and female–male sets. None of that information was available at the time of our analyses.

We were led to our hypothesis by theoretical and empirical literature arguing that natural selection conserved biological mechanisms that manifest in reproductive suppression, brood reduction, offspring sex selection and selection *in utero*. We and others have argued that this literature leads to the prediction of fewer than expected twins among male births in stressed populations. Other literature may also lead to the prediction and suggest other lines of further research. Work describing competition between twins for resources *in utero* [55] could, for example, lead to the argument that the maternal stress response somehow affects the intensity and outcome of the competition. We, however, know of no such speculation in the literature.

Although multiple studies have supported the argument that twins will appear less frequently than expected among males born to stressed populations, the stressors tested vary widely in their nature as do the reported effect sizes. No work has yet attempted to array the stressors *a priori* on any dimension of dose. If this were possible, future research could test the intuitively appealing argument that differences in the strength of association reflects not only differences in the characteristics of the populations tested, but also in the dose of stress they likely suffered.

We cannot claim that our findings have clear clinical implications although they suggest increasing, during stressful times, public health programming focused on women of reproductive age. Our findings do, however, have implications for our understanding of how evolution affects the nature and experience of contemporary populations. They call attention to the facts that selection *in utero* shapes the characteristics of human birth cohorts and that collective as well as individual choices likely affect the targets and depth of that selection [56]. They, moreover, explicitly connect those facts to the pandemic. The threat or experience of infection, the cascade of stressors entailed by pandemic restrictions, the loss of access to social resources, and the constraint of restoration all likely affect selection *in utero*.

**Conflict of interest:** None declared. 
